# Better to Be in Bad Company than to Be Alone? *Aedes* Vectors Respond Differently to Breeding Site Quality in the Presence of Others

**DOI:** 10.1371/journal.pone.0134450

**Published:** 2015-08-05

**Authors:** Thais I. S. Riback, Nildimar A. Honório, Renato N. Pereira, Wesley A. C. Godoy, Cláudia T. Codeço

**Affiliations:** 1 Programa de Computação Científica, Fundação Oswaldo Cruz, Rio de Janeiro, Rio de Janeiro, Brazil; 2 Laboratório de Transmissores de Hematozoários, Instituto Oswaldo Cruz, Fundação Oswaldo Cruz, Rio de Janeiro, Rio de Janeiro, Brazil; 3 Instituto de Ciências Exatas, Universidade Federal Rural do Rio de Janeiro, Seropédica, Rio de Janeiro, Brazil; 4 Departamento de Entomologia e Acarologia, Escola Superior de Agricultura Luiz de Queiroz, Universidade de São Paulo, Piracicaba, São Paulo, Brazil; University of Tours, FRANCE

## Abstract

This study focuses on two competing species, Aedes aegypti and Aedes albopictus (Diptera: Culicidae), both invasive mosquitoes of the New World. Context-specific competition between immature forms inside containers seems to be an important determinant of the coexistence or displacement of each species in different regions of the world. Here, competition experiments developed at low density (one, two or three larvae) and receiving four different resource food concentration, were designed to test whether Ae. albopictus and Ae. aegypti respond differently to competition, and whether competition can be attributed to a simple division of resources. Three phenotypic traits - larval development, adult survival under starvation and wing length - were used as indicators of performance. Larvae of neither species were limited by resource concentration when they were alone, unlike when they developed with competitors. The presence of conspecifics affected Ae. aegypti and Ae. albopictus, inducing slower development, reduced survival and wing length. The response to resource limitation was different when developing with heterospecifics: Ae. aegypti developing with one heterospecific showed faster development, producing smaller adults with shorter lives, while in the presence of two competitors, development increased and adults lived longer. Aedes albopictus demonstrated a better performance when developing with heterospecifics, with no loss in their development period and improved adult survival. Overall, our results suggest that response to competition can not simply be attributed to the division of resources, and that larvae of both species presented large phenotypic plasticity in their response to the presence or absence of heterospecifics and conspecifics.

## Introduction

This study focuses on two competing species, *Aedes aegypti* and *Aedes albopictus* (Diptera: Culicidae), both invasive mosquitoes of the New World [1]. Context-specific competition between *Ae*. *aegypti* and *Ae*. *albopictus* immature forms—which takes place inside natural and/or artificial larval breeding sites varying in size, shape and food resource availability,—seems to be an important determinant for the *Ae*. *aegypti* exclusion in Florida [2–3], the coexistence of both species in Brazil, Cameroon and Nigeria [4–6] and the *Ae*. *albopictus* exclusion in Leticia, Colombia [7]. Mechanisms of mating interference of *Ae*. *albopictus* males on *Ae*. *aegypti* females causing the sterilization of the latter, have also been proposed to explain the spatial segregation of these species in some areas [8–9]; beyond this, sharing space with other container dwelling species may also leads to competition, predation and other types of ecological interactions, and this situation leads to a limitation in the amount of available food [10–11].

Many studies have investigated the environmental factors associated with the different competition outcomes, looking at life history traits. Life history theory seeks to explain organism evolutionary characteristics such as adaptive response to environmental variation, differences in mortality or resource allocation in life stages, also examining how they are correlated and influenced by ecological factors [12–13]. The resource availability during initial life stages [14], environmental stress degree, as well as resource competition [15], greatly influences individual life history traits [12,16–17], such as metamorphosis, maturation, and reproduction; the population’s success can also be influenced by the timing of these events and by the overall condition of the organisms [18–20].

Differences in the detritus type [21–22] and the quality of food resources [23], can produce differences in larval foraging behavior, being observed that *Ae*. *albopictus* presented best performance when growing in environments containing vegetal food resources, while a similar performance was observed for *Ae*. *aegypti* developing in the presence of animal derived resources [10,21,24]. Furthermore, larval density [25–29], the composition of insect's microbiota [30], dissolved oxygen concentration [31] as well as the presence of toxic substances dissolved in water [32–33] also affect the number of eggs hatched, larval size, development time, adult size, longevity and fecundity [10–11,26,34–36].

Classically, mosquito larvae competition experiments are set up with a large number of larvae per vessel, varying between 40 and 400 [25–29]. Studies developed inside cemetries areas, where it is common find out artifical larval breeding sites such as flower pots that vary in size, shape and water volume, it is possible observe a smaller number of larvae in comparison with that used in competition experiments [37–38]. As deaths and metamorphosis occur at different times, density conditions are not kept uniform; consequently the life history parameters of surviving individuals are likely to be affected by their specific conditions. Bedhomme et al. [36] developed a precisely controlled experimental setup to study the reaction of several life history parameters of *Ae*. *aegypti* in response to larval competition. They were interested in the reaction norms of males and females to intraspecific resource competition. Here, we revisit Bedhomme's [36] experimental design, in order to investigate the response of *Ae*. *aegypti* and *Ae*. *albopictus* immatures to competition.

Following this protocol, one, two or three larvae per vessel are used, and the questions posed are whether *Ae*. *albopictus* and *Ae*. *aegypti* respond differently to competition, and whether competition can be attributed to a simple division of resources. More specifically, the following questions were addressed: Are both species equal competitors? Are there differences in the effects of competition on life history traits? Can competition be simply attributed to a division of resources?

## Material and Methods

Ethics Statement: No specific permits where required for the described laboratory studies. The present work was a laboratory study developed with eggs of *Ae*. *aegypti* and *Ae*. *albopictus*, both mosquitoes species introduced in the Americas from Africa and Asia, and are important vectors of dengue, chikungunya and yellow fever virus. These are not endangered or protected species; adults of both species were collected in open areas where were not necessary specific permissions to collect it, the specimens were brought to laboratory and kept at colonies where eggs were collected.

### General procedures

Experiments were performed with *Ae*. *aegypti* and *Ae*. *albopictus* eggs from a laboratory open colony established from specimens collected in Rio de Janeiro-RJ/Brazil. Newly hatched larvae were transferred to Petri dishes, and received 6mg (first day) and 8mg (second day) of fish food (Tetramin) dissolved in 1ml of water/mg/larva [36]. After 48 hours, sets of 1, 2 or 3 larvae were transferred to 30ml plastic Beckers filled with 5ml of water containing fish food dissolved at different concentrations (see below). The fish food solution was replaced daily, in order to avoid detritus accumulation and to provide a stable resource environment for the larvae.

The experimental units were kept in a Biochemical Oxygen Demand chamber (B.O.D.) at 25°C ± 1°C, and were observed every 24 hours in order to record dates of pupation, emergence and death of adults. The pupae were individually transferred to vials containing 4ml of water until adult emergence, which were kept in the same vials without water, in order to avoid drowning, only receiving water from a soaked cotton [36]. After death, adults were individually transferred to 1.7ml plastic vials and kept frozen. Wing measurements were done by extracting the right wing (left wings were used only if the right wing was damaged) from each adult and measuring the distance between the allula notch and the tip of the wing [39], using a dissecting microscope fitted with a graduated eyepiece.

### Experiment

#### Limiting food concentration for larval development

Solutions with 1mg, 2mg, 4mg, 8mg, 16mg, 32mg and 64mg of fish food dissolved in 5ml of water were offered and replaced daily to 15 individually separated *Ae*. *aegypti* and *Ae*. *albopictus* larvae. Survival was assessed daily. Both species presented an increased mortality at the three highest concentrations (*Ae*. *aegypti*—16mg: 6.6%, 32mg: 80%, 64mg: 93.3%; *Ae*. *albopictus*—16mg: 40%, 32mg: 40%, 64mg: 86.6%), possibly due to the organic matter decomposition leading to the formation of a film on the water surface. In order to avoid this effect, subsequent experiments were performed using 1mg, 2mg, 4mg and 8mg of fish food dissolved in 5ml of water.

#### Density combination x Resource concentration

The experiment followed a factorial design with two factors. The first factor has six larval combinations, named A to F (Table 1):

(Alone) to measure life history traits in the Absence of competitors: experiment consisted of—a single larva (experiment A), being either a single larva of *Ae*. *aegypti* or *Ae*. *albopictus*;

(Intra) to test Intra-specific competition: a larva of either species growing with 1 conspecific larva (B) or with 2 conspecific larvae (C);

(Inter) to test inter-specific competition—a larva of either species growing with 1 heterospecific larva (D); with 1 conspecific and 1 heterospecific larvae (E); or with 2 heterospecific larvae (F).

**Table 1:** Larvae density treatment used during the laboratory experiments performed with *Ae*. *aegypti* and *Ae*. *albopictus*.

Density treatment		Larval combination
No competitors	A	Larva alone
Intra-specific competition	B	Larva + 1 conspecific
	C	Larva + 2 conspecific
Inter-specific competition	D	Larva + 1 heterospecific
	E	Larva + 1 conspecific + 1 heterospecific
	F	Larva + 2 heterospecifics

The second factor in the experimental design is “resource” with the four concentrations described above. The competition experiment consisted of 6 density combinations x 4 food concentrations x 15 replicates, totalizing 360 experimental units.

This protocol contrasts with most competition experiments, in which tens of larvae grow in the same vial. The advantages of using such minimal experiment are the larger number of replicates, and the control of density conditions through the whole experiment [36, 40].

### Statistical analysis

#### Life history traits investigated

Larval development time was measured from egg hatching to pupation while adults survival was measured from emergence to death under starvation. Both traits were measured in days. The third trait measured was wing length, in mm. In order to ensure that all replicates had the nominal density during the whole study, only those where all larvae reached pupation were retained for statistical analysis [36]. Besides, only one individual from each replicate was randomly selected to be used during the statistical analysis, thus avoiding pseudo-replication. This individual is referred to as the “target larva” [41].

#### Testing the effects of densities and food concentration on life history traits

Differences in life history traits between sexes of both species were tested by the Kolmogorov-Smirnov test, with significant results for wing length and adult survival. Considering that the number of replicates was not sufficient to analyze both sexes separately, we opted to standardize and pool the female and male measurements by subtracting the observed values from the corresponding sex-specific means, obtaining centralized variables. Thus the response variables considered for further analyses were: development time, centralized wing length, and centralized adult survival. First, we assessed the effect of food concentration on each of the three traits of *Ae*. *aegypti* and *Ae*. *albopictus* growing in the single larva experiment using one-way ANOVA (wing length and survival) and Kruskal-Wallis tests (developmental time). The latter did not attend the normality assumptions. Secondly, we carried out two-way ANOVAs for each trait having food concentration (4 levels) and competition type as factors. The competition type factor was considered in different ways in order to test the study hypotheses, as more clearly presented in the section below on contrasts). For each model, we tested the normality and homogeneity of residuals and in the presence of deviation from normality, we reanalyzed the model using power transformed data. In general, wing length never required transformation while survival and developmental time required transformation to the powers varying from -0.9 to 1.3. The results of these models did not differ qualitatively from those using the untransformed data and for simplicity, just these are presented. The effect of treatments on adult survival was further investigated with the non-parametric Kaplan-Meier and Log-rank tests.

#### Testing the cost of sharing breeding site with conspecifics or heterospecifics

Three contrasts were defined (Table 2) and tested by Scheffé's test in order to evaluate two specific hypotheses. The first one was whether changes in life history parameters are expected by simply increasing larval density, disregarding the type of species, expressed in the form of two contrasts:

*Contrast I*: comparing a larva growing alone versus larva growing with another larva;

*Contrast II*: comparing a larva growing alone versus larva growing in the presence of two other larvae.

**Table 2:** Contrasts analyzed between the six different treatment densities combination. Analysis were performed for *Ae*. *aegypti* and *Ae*. *albopictus* and the four food concentration offered.

Contrast	Density treatment combinations	Description
**I**	A x (B + D)	Individual A) alone compared with the one developed in the presence of one B) intra-specific or D) inter-specific competitor
**II**	A x (C + E + F)	Individual A) alone compared with the one developed in the presence of two C) intra-specific, E) both or F) inter-specific competitors
**III**	(B + C) x (D + F)	Individual developed in the presence of one or two competitors B,C) intra-specific, D,F) inter-specific

The second one was whether changes in life history parameters are expected when larvae developed with conspecifics (at any density) or with heterospecifics (at any density), expressed by *Contrast III*: comparing larvae developed at intra-specific versus inter-specific interactions. In the presence of a significant contrast, further analysis was conducted using Tukey's test, with the purpose of identifying which treatments were responsible for the significance. Analyses were performed separately for each species. All statistical analyses were performed using the software R, version 3.0.2 [42].

## Results

### General results

*Aedes aegypti*: Larval development time was similar between the sexes (M = 6.48±1.08 days and F = 6.84±1.13 days; D = 0.1274, *P* = 0.1525). Males lived longer (M = 8.49 ± 2.47 and F = 6.74± 2.38 days; D = 0.33, *P*<0.001), and presented shorter wings (M = 2.44 ± 0.2 and F = 3.11 ± 0.26 mm; D = 0.87, *P*<0.001).

*Aedes albopictus*: larval development time (M = 6.16±0.93 days and F = 6.5±1.01 days; D = 0.12, *P* = 0.2186), and adults survival were similar between the sexes (M = 5.11±2.12; F = 5±2.17 days; D = 0.0794, *P* = 0.7186); males presented shorter wings (M = 2.34 ± 0.2 and F = 2.82 ± 0.21 mm; D = 0.7438, *P*<0.001).

### Testing the effects of densities and food concentration on life history traits

#### Growing in the absence of competitors—Treatment A (Table 1)

The impact of resource concentration on life history traits was analyzed by the Kruskal-Wallis test.

**Fig 1:**
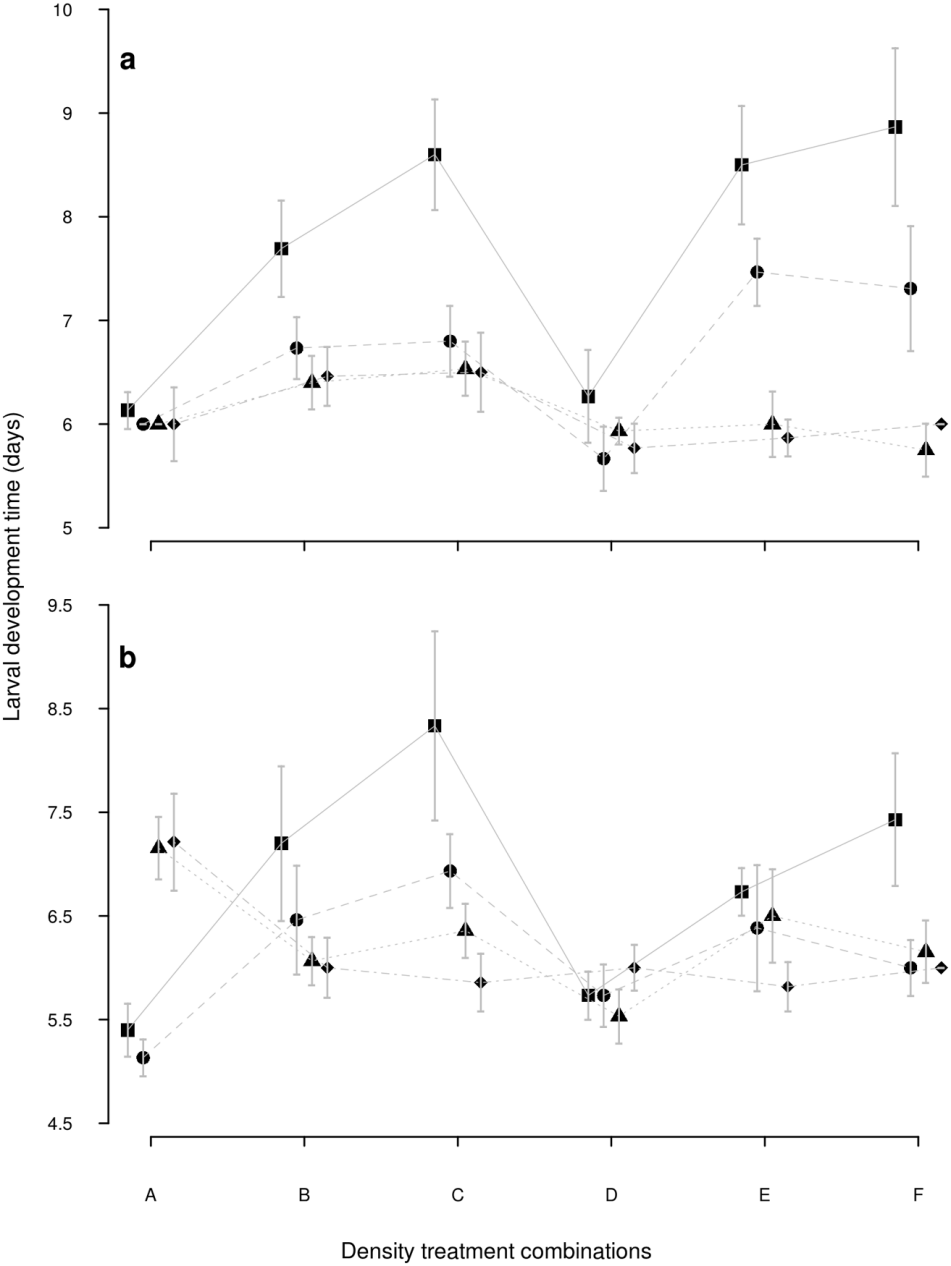
Larval development time of *Aedes aegypti* and *Aedes albopictus*. Larval development time (Mean±SE) of a) *Aedes aegypti* and b) *Aedes albopictus* developed at six different larval density combinations (A: individual alone; B: Larva + 1 conspecific; C: Larva + 2 conspecific; D: Larva + 1 heterospecific; E: Larva + 1 conspecific +1 heterospecific; F: Larva + 2 heterospecifics) and four food levels (1mg: square; 2mg: circle; 4mg: triangle; 8mg: diamond).

**Fig 2:**
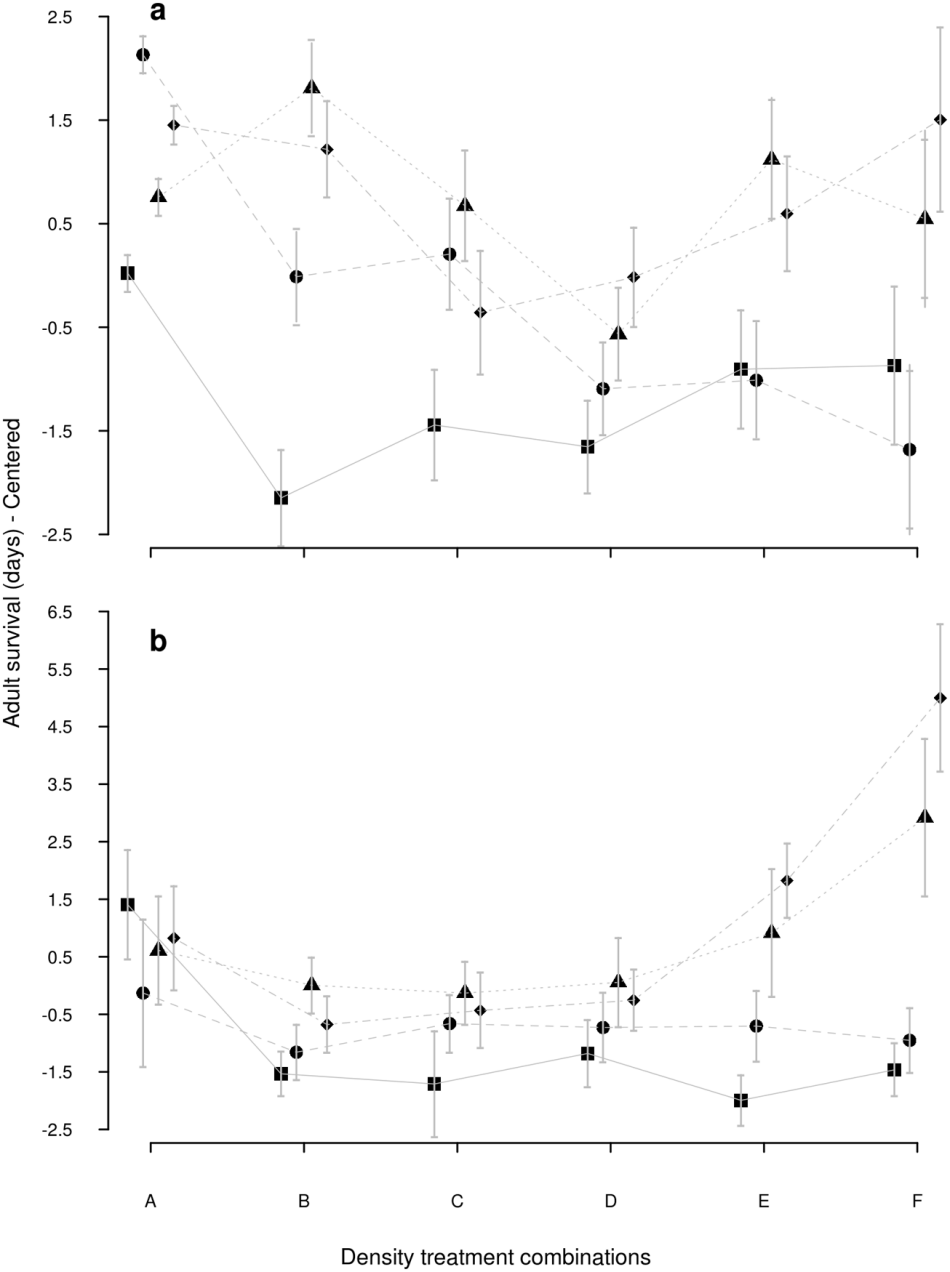
Adult survival at starvation of *Aedes aegypti* and *Aedes albopictus*. Adult survival at starvation (days)–Centered results (Mean±SE) of a) *Aedes aegypti* and b) *Aedes albopictus* developed at six different larval density combinations (A: individual alone; B: Larva + 1 conspecific; C: Larva + 2 conspecifics; D: Larva + 1 heterospecific; E: Larva + 1 conspecific +1 heterospecific; F: Larva + 2 heterospecifics) and four food levels (1mg: square; 2mg: circle; 4mg: triangle; 8mg: diamond).

**Fig 3:**
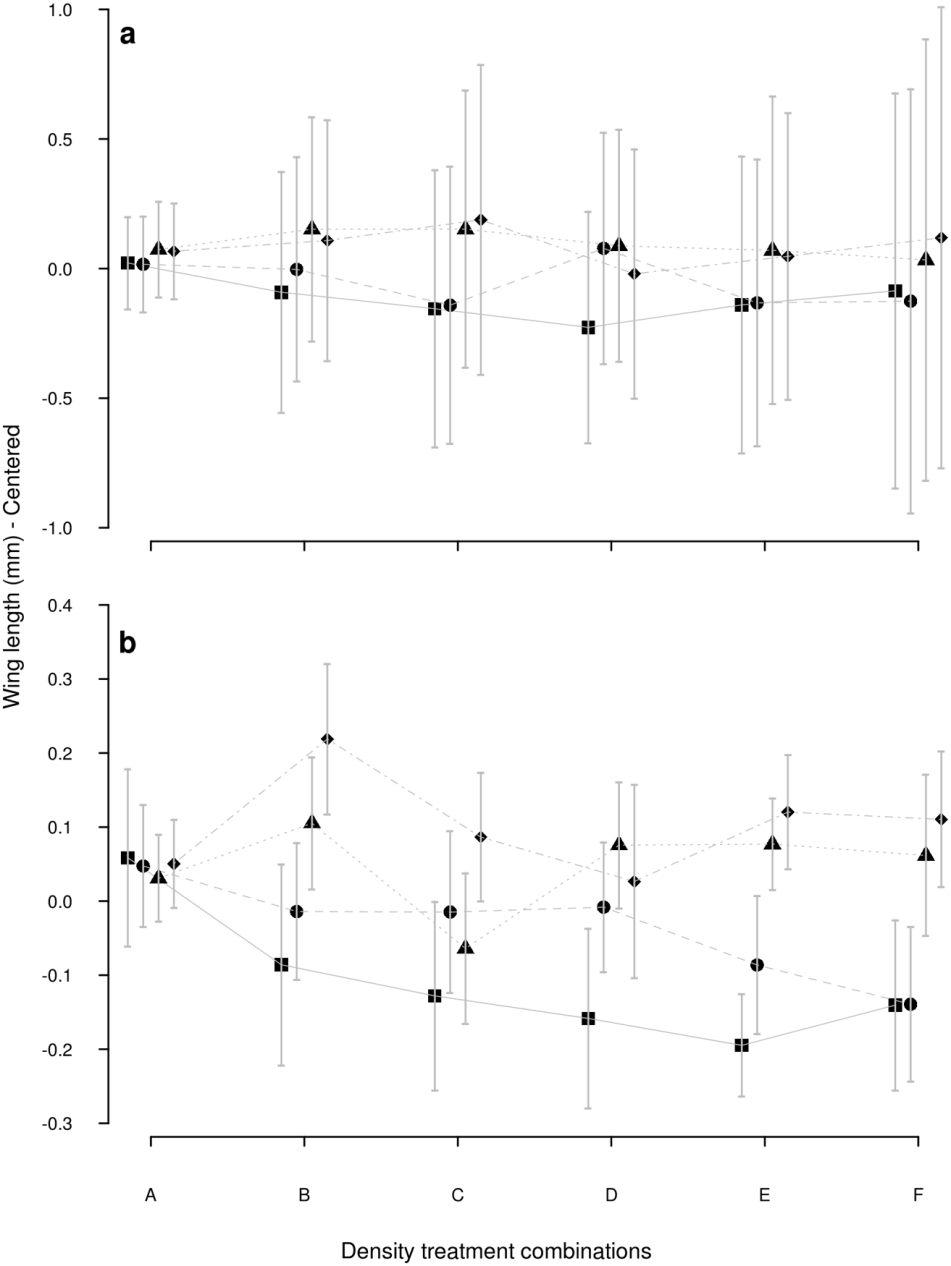
Wing length of *Aedes aegypti* and *Aedes albopictus*. Wing length (mm)–Centered results (Mean±SE) of a) *Aedes aegypti* and b) *Aedes albopictus* developed at six different larval density combinations (A: individual alone; B: Larva + 1 conspecific; C: Larva + 2 conspecifics; D: Larva + 1 heterospecific; E: Larva + 1 conspecific +1 heterospecific; F: Larva + 2 heterospecifics) and four food levels (1mg: square; 2mg: circle; 4mg: triangle; 8mg: diamond).

*Aedes aegypti*: larval development time (Fig 1a—6.035 ± 0.37 days, *X* ^*2*^ = 1.38, *P* = 0.71) and adult survival were marginally affected by resource concentration (Fig 2a—*X* ^*2*^ = 7.18, *P* = 0.067). Wing length (Fig 3a) was slightly smaller at the lower food concentrations, but this difference was not significant (*X* ^*2*^ = 1.78, *P* = 0.61).

*Aedes albopictus*: larval development time (Fig 1b) was affected by food concentration, being faster (5.27 ± 0.44 days) at the two lowest concentrations, and slower (7.18 ± 0.73 days) at the two highest concentrations (*X* ^*2*^ = 45.94, *P<*0.001). Adult survival (Fig 2b) was slightly higher at the lowest concentration but not significantly (*X* ^*2*^ = 3.71, *P* = 0.29). Wing length (Fig 3b, *X* ^*2*^ = 0.19, *P* = 0.98) was not affected by the resources offered.

#### Growing in the presence of one competitor—Treatments B (1 conspecific) and D (1 heterospecific) (Table 1)

*Aedes aegypti*: resource concentration contributed to explain 16 to 18% of the variation of each of the three life history traits. Competitor type contributed to explain ca. 30% of the larval development variation and less than 5% of the other two traits (Table 3). Larvae took longer to reach pupation (7.69±0.85 days) when developed in the presence of conspecifics, and the period was shortened as food availability increased. In the presence of heterospecific competitors, the rate of development was not delayed (Fig 1a). This differential development in the presence of competitors had a slight but significant effect on adult survival (Fig 2a), which lived longer as the food limitation relaxed, and this improvement was greater for individuals from the conspecific treatment (survival analysis presented similar pattern (S1 Fig Line 1)). Wing length also improved as the food limitation relaxed, but it was not influenced by the type of competitor (Fig 3a).

**Table 3:** Summary of ANOVA results for the effects of food resources and density treatment combinations on larval development time, adult survival and wing length transformed, for *Ae*. *aegypti* and *Ae*. *albopictus*.

Source of Variation	Development time (days)						Adult survival (days)						Wing length (mm)					
	*Ae*.*aegypti*			*Ae*. *albopictus*			*Ae*.*aegypti*			*Ae*. *albopictus*			*Ae*.*aegypti*			*Ae*. *albopictus*		
**One Competitor**	df	SS (%)	*P*	df	SS (%)	*P*	df	SS (%)	*P*	df	SS (%)	*P*	df	SS (%)	*P*	df	SS (%)	*P*
Resource	3	16.72	<0.001	3	7.74	0.008	3	17.53	<0.001	3	20.03	<0.001	3	18.29	<0.001	3	18.25	<0.001
Competitor	1	29.14	<0.001	1	15.39	<0.001	1	4.71	0.013	1	1.57	0.161	1	1.4	0.201	1	2.35	0.087
Resource X Competitor	3	4.71	0.021	3	8.69	0.004	3	4.46	0.114	3	0.44	0.904	3	3.17	0.297	3	2.52	0.363
Residuals	106			107			100			99			91			98		
**Two Competitors**	df	SS (%)	*P*	df	SS (%)	*P*	df	SS (%)	*P*	df	SS (%)	*P*	df	SS (%)	*P*	df	SS (%)	*P*
Resource	3	60.62	<0.001	3	30.76	<0.001	3	10.97	<0.001	3	37.51	<0.001	3	21.79	<0.001	3	22.41	<0.001
Competitor	2	0.169	0.694	2	5.08	0.0013	2	0.036	0.967	2	13.01	<0.001	2	0.58	0.568	2	0.01	0.98
Resource X Competitor	6	3.789	0.015	6	9.03	<0.001	6	5.04	0.184	6	15.1	<0.001	6	2.06	0.67	6	4.62	0.18
Residuals	153			151			149			145			148			144		

*Aedes albopictus*: larval development was affected by both resources and competitor type, while adult survival and wing length were affected by resource amount only (Table 3). Resource concentration was the main effect explaining variation in survival and wing length (20% and 18%, respectively, (Table 3)). Competitor type, on the other hand, explained 15% of the larval development variation but less than 2% of the other traits. When kept in the presence of a conspecific, it was observed a gradual reduction in the development time as resource increased; this pattern was not observed when growing with a heterospecific (Fig 1b).

The presence of a conspecific induced a shorter development time when resource was abundant and a long development time when resource was scarce (Fig 1b). Mosquitoes from the low diet tended to be smaller and more fragile and, even when diet was abundant, survival was not as high as in the single larva treatment, while wing size was actually greater than in the single larva treatment (Figs 2b and 3b). The presence of the heterospecific was an important factor inducing fast *Ae*. *albopictus* development (Fig 1b); (survival analysis presented similar pattern (S2 Fig—Line 1)). The resulting adults presented features that were similar to the conspecific treatment. The only difference was that mosquitoes were not as big as in the conspecific scenario.

#### Growing in the presence of two competitors—Treatments C (2 conspecific), E (1 conspecific and 1 heterospecific) and F (2 heterospecifics) (Table 1)

*Aedes aegypti*: all life history traits were significantly affected by the resource concentration (larval development: 60%; wing length: 21%; adults survival: 10%), while competitor type was less influential in these 3-larvae treatments (Table 3). In general, larvae presented longer development time when at the lowest food concentrations, slightly more in the presence of heterospecific larvae (Fig 1a). The mean survival time of individuals who developed with one (Treatment E) or two heterospecifics (Treatment F) increased with food concentrations (Fig 3a); (survival analysis presented similar pattern (S1 Fig—Line 2)).

*Aedes albopictus*: it was strongly affected by resource concentration since larval development was slower as resource became scarcer, adult survival was lower and wings were shorter (Figs 1b, 2b and 3b; Table 3). Competitor type and resource x competitor type interaction were significant factors for development and adult survival, but not for wing length (Table 3). Development time presented the largest variation when the competitor was a conspecific, while the presence of a heterospecific induced a homogenization (Fig 1b). The two lowest resource treatments negatively affected adult survival and wing length, regardless of the competitor type (Figs 2b and 3b). However, *Ae*. *albopictus* presented unexpected improvement of adult survival when growing with heterospecifics at higher food concentration (Fig 2b); (survival analysis presented similar pattern (S2 Fig—Line 2)). On the other hand, it was possible to observe longer wings in individuals developing with a conspecific rather than with a heterospecific (Fig 3b).

### Testing the cost of sharing breeding site with conspecifics or heterospecifics

#### Life history parameters versus larval density (Contrasts I and II, Table 2)

*Aedes aegypti*: was little affected by larval density variation; contrast I (Ax(B+D); Table 2) revealed that adults from 2mg developing in the presence of any type of competitor presented shorter survival than those kept alone (Fig 2a). Contrast II (Ax(C+E+F); Table 2) revealed that larvae kept with any type of competitor and at the lowest food concentration, presented an increase in their development time (Fig 2a, Table 4), while adults developing with heterospecifics (treatment E and F), and receiving 2mg of food presented shorter survival (Fig 2a, Table 4).

**Table 4:** Summary of Tukey analysis performed for density treatment combinations for *Ae*. *aegypti* and *Ae*. *albopictus*.

***Aedes aegypti***									
Contrast	Density treatment combination	Larval development (days)				Adult survival (days)			
		1mg	2mg	4mg	8mg	1mg	2mg	4mg	8mg
I	Alone (A) x Intra1 (B) + Inter1 (D)	-	-	-	-	-	***	-	-
II	Alone (A) x Intra2 (C) + Both (E) + Inter2 (F)	***	***	-	-	-	**	-	-
III	Intra1 (B) + Intra2 (C) x Inter1 (D) + Inter2 (F)	-	-	***	***	-	-	-	-
***Aedes albopictus***									
Contrast	Density treatment combination	Larval development (days)				Adult survival (days)			
		1mg	2mg	4mg	8mg	1mg	2mg	4mg	8mg
I	Alone (A) x Intra1 (B) + Inter1 (D)	-	***	***	***	***	-	-	-
II	Alone (A) x Intra2 (C) + Both (E) + Inter2 (F)	***	***	***	***	***	-	-	-
III	Intra1 (B) + Intra2 (C) x Inter1 (D) + Inter2 (F)	-	-	-	-	-	-	***	***

***: *P* < 0.001.

**: *P* < 0.05.

'—': Tukey analysis was not performed for the contrasts which did not show any differences among the larval competition combinations.

*Aedes albopictus*: larval development was significantly affected by density at all resource concentrations according to Contrasts, I and II (Table 2). Larvae kept alone presented shorter development period at the two lowest food concentrations (1 and 2 mg) whereas the opposite was observed at 4 and 8mg (Fig 2a, Table 4). Survival of the emerged adults was affected by larval density as well (Fig 2b, Table 4), but only at the most limiting resource concentration (1mg).

#### Life history traits of individuals growing with conspecifics versus heterospecifics (Contrast III, Table 2)

*Aedes aegypti*: larval development was significantly affected by the presence of heterospecifics (treatment D and F) at the two highest food concentrations, but neither their adult survival nor their wing length were affected (Fig 2a, Table 4).

*Aedes albopictus*: development time was not significantly affected by competitor type (Table 4). Adult survival, on the other hand, was significantly different at the highest food concentrations (Table 4). It is interesting to note that survival increased as the number of heterospecific larvae (Treatment F, Table 2) increased (or, in other words, the number of conspecific larvae decreased) (Fig 2b). Wing length was not significantly affected by the competitor type (Table 3).

## Discussion

### The effects of densities and food concentration on life history traits

Life history traits are greatly influenced by the environmental conditions to which individuals are exposed, but such expressions do not occur in an isolated way; in fact, body size can be a reflection of development time according to the environmental conditions imposed, and adult survival may also be influenced by body size [43]. Presumably, a short development time is supposed to be beneficial for species with opportunistic life-cycles, especially if they occupy transient environments, as is the case of the majority of artificial containers. The advantage of a shorter development time is to reduce the risk of dying before reproduction and to increase the number of generations per season [13].

On the other hand, a longer development time may be required to achieve an optimal size at maturity. Classical life history models postulate that organisms that show adaptive plasticity in their development time, are supposed to delay their metamorphosis when conditions are good and to metamorphose as soon as they achieve the minimum size, when conditions are poor [44]. The underlying assumption of this model is a constant growth rate, and the outcome is a direct relationship between development time and adult size. Furthermore, it is known that factors such as presence of competitors [45–46] and resource quality [47] can negatively influence mosquitoes’ life history, producing smaller and shorter-living adults.

*Aedes aegypti* development time was strongly affected by resource limitation (Table 3) and this result is in agreement with the literature. A recent study using meta-analysis by Couret and Benedict [48] shows that, after temperature, larval density is the main factor affecting *Ae*. *aegypti* larval development in laboratory and field experiments. When growing alone, on the other hand, this species showed invariable development time and similar adult traits, regardless of the food concentration provided and these characteristics, typical of *Ae*. *aegypti* fed *ad libitum*, suggest that resource concentrations were not limiting for a larva growing alone [49].

Resource concentration was more important than competitor type for *Ae*. *aegypti* adults’ survival and wing length. Adult survival under starvation presented a similar pattern between experiments, with increased survival as the availability of food increased; however, wing length was not affected. When comparing individuals which developed at the presence of competitors with the alone condition, competition environment presented a negative effect, causing a slight reduction at the adults survival, especially when kept with conspecifics. These results suggest that *Ae*. *aegypti* larvae may tolerate the environmental stress through a better efficiency in sharing available resources [47], leading to increased survival and wing length, even when growing with competitors.

Interestingly, *Ae*. *albopictus* showed large variation in development time when growing alone, which was not followed by variation in wing length or survival. In particular, when resources were abundant, metamorphosis was delayed but that delay did not produce bigger or long lived adults. It is possible that the high food concentration was toxic for *Ae*. *albopictus* larvae but, if so, no further effect was detected at the adult stage. Another possible explanation for the delayed development at high food concentration could be a behavioral response in a non-risky environment (absence of predators or competitors).

*Aedes albopictus* adults were homogeneous in size when growing alone, but this homogeneity was shaken by the introduction of a competing larva. At low food concentrations, mosquito size was reduced, suggesting resource limitation. But when food was more abundant, wing length actually increased, in comparison with the 1 larva treatment. Survival was never as good as when the larva was alone, but it improved as resource availability increased as well. These results suggest that intraspecific competition has an impact on development, survival and wing length, as described in the literature. Moreover, researchers also suggest that the presence of a conspecific larva stimulates development, potentially resulting in larger animals. This is not explained by resource division alone.

### The cost of sharing breeding site with conspecifics or heterospecifics

As the number of larvae in the vessels increased, resource limitation at the lowest food concentration was evident, affecting all life history traits; these effects depended on the competitor type. The presence of conspecifics induced slow development and reduced survival in *Ae*. *aegypti* and *Ae*. *albopictus*. Resource limitation seemed to occur at the low food treatment when one larva was present, while the high food treatment became limiting only with the presence of three larvae. Wing length tended to decrease as the number of conspecific competitors increased, although not significantly. These results are in accordance with the expected effects of intraspecific competition on *Ae*. *aegypti* and *Ae*. *albopictus* phenotypes [36,40,50].

Contrary to the intraspecific situation (when the presence of a conspecific delayed development), *Ae*. *aegypti* larvae were actually faster when developing with one heterospecific than when they were alone. As a result, the adults originated from this condition tended to survive less than in the presence of a conspecific. Wing length was also smaller at the lowest concentration. Faster development of *Ae*. *aegypti* growing in the presence of a heterospecific, combined with poor life history traits, suggests that fast development was a response to a stressor with a cost. Furthermore, the fact that adult survival varied more than wing length suggests that the former is a more plastic trait, that is, in the case of facing malnutrition, the impact occurs first on reserves rather than on structure. When competing with two heterospecifics, *Ae*. *aegypti* larvae development was longer, taking nine days at the lowest food concentration. The emerging adults lived as much as the one larva competitor condition, and both of them were worse than in the single larva treatment. These results suggest that the presence of two larvae at low food concentration was stressful, while the stress imposed by one larva depended on whether it was an *Ae*. *albopictus* or not. Experiments performed by Ho et al. [26] presented a similar pattern, where *Ae*. *aegypti* growing with *Ae*. *albopictus* or with *Aedes triseriatus* took less time to reach pupation and wing length reduction, suggesting that stress was occurring and was caused by the presence of heterospecific competitors.

As food availability increased, resource limitation relaxed and development time was reduced to six days, when in the presence of heterospecifics, and it was reduced to 6.5 days in the presence of conspecifics. Again, the presence of *Ae*. *albopictus* stimulated faster larval development. No evident cost was detected for this fast development, neither in terms of survival nor wing length, suggesting that *Ae*. *albopictus* is a source of stress for *Ae*. *aegypti* even at abundant food concentrations, and responded by increasing its rate of development. If there was any cost for the adult traits, it was not detected in the experiment.

*Aedes albopictus* larval development tended to be slower in the presence of a conspecific rather than of a heterospecific, and survival was improved in the presence of heterospecifics. These results do not support the idea that *Ae*. *aegypti* is a stressor for *Ae*. *albopictus*, because adult traits were actually better in the presence of *Ae*. *aegypti* than in the presence of a conspecific. Similar patterns of development time and adult survival were observed by Constanzo et al. [50] in competition experiments performed with *Ae*. *albopictus* and *Culex pipiens*, developing at different resource food concentrations. One possible explanation is that *Ae*. *albopictus* presented better adaptability to unfavorable conditions considering that, in the presence of two competitors of any species, development time was faster when kept at the lowest food concentration, while it reached pupation together with *Ae*. *aegypti* at the two highest food concentrations.

Previous studies have shown the association between resource limitation and slower development, shorter survival under starvation and smaller adult size of *Ae*. *aegypti* and *Ae*. *albopictus* in intra- and interspecific competition experiments [36,40,50–51]. Results observed in the present work suggest an asymmetry in the response of *Ae*. *aegypti* and *Ae*. *albopictus* to competition. It is important to consider that these responses are context-specific and likely to be affected by the type of detritus used.

Taking into account that survival under starvation is a measurement of nutrient reserves, and that these reserves are related to the quality and quantity of food resources available in a container and consumed by larvae during development [43], the results observed in the present work suggest that the stress response of both *Ae*. *aegypti* and *Ae*. *albopicus* involved changing their allocation of energy between reserve and structural tissues. The complexity of behaviors originated from the different combinations of density and food, a situation that could be easily observed in the field, suggests that *Ae*. *aegypti* and *Ae*. *albopictus* are not equal competitors, responding differently to environmental conditions through differences in their life history traits.

### *Aedes albopictus*: a closer look

Differences between the results for *Ae*. *albopictus* in the present work and those described in the literature trigger a few questions about the life history traits considered. How sensitive are the larval development time and adult survival to the different number of competitors and by the kind of food resources used in the various competition experiments published in the literature? Could the competition superiority observed during the larval stage have costs in terms of the adult phenotype (lower survival)? *Aedes albopictus* presented larval development time and adult survival similar to the ones observed by Constanzo et al. [50] when studying the effects of intra and interspecific competition with *Cx*. *pipiens*, presenting reduction on development time and larger adult survival when developed with heterospecifics. Could this behavior, especially in relation with adult survival, suggest that these are species-specific behaviors?

The results on behavior differences indicate the necessity of developing competition studies that take into account the foraging behavior and survival capacity of each species, trying to find a balance between the types of food available. That would approximate the situations observed in the field, where there would be a higher chance of finding leaves and decomposed animals in natural containers. A more detailed investigation of the survival of adults emerging from different larval development conditions is also necessary, considering the number of individuals and resource availability in a container, in order to observe whether there are specific patterns of survival regardless of condition that were imposed to the larvae, or whether species have some kind of plastic response, that allows adjusting to the conditions to which they were submitted at a given time. At last, it is important to note that these experiments were done with *Aedes* populations from Brazil, and further studies should be done to verify if these findings would be similar for *Aedes* populations from temperate regions, such as USA or Argentina.

## Supporting Information

S1 Database 'Data_Competition_Aedes.csv'. Database collected during the experimental period and used to perform all statistical analysis.
(CSV)

S1 Fig *Aedes aegypti* survival analysis. *Aedes aegypti* survival analysis—Line 1: comparison between individuals kept alone (A: individual alone) with those kept at the presence of one competitor (B: Larva + 1 conspecific; D: Larva + 1 heterospecific). Line 2: comparison between individuals kept alone (A: individual alone) with those kept at the presence of two competitors (C: Larva + 2 conspecifics; E: Larva + 1 conspecific +1 heterospecific; F: Larva + 2 heterospecifics).
(TIF)

S2 Fig *Aedes albopictus* survival analysis. *Aedes albopictus* survival analysis—Line 1: comparison between individuals kept alone (A: individual alone) with those kept at the presence of one competitor (B: Larva + 1 conspecific; D: Larva + 1 heterospecific). Line 2: comparison between individuals kept alone (A: individual alone) with those kept at the presence of two competitors (C: Larva + 2 conspecifics; E: Larva + 1 conspecific +1 heterospecific; F: Larva + 2 heterospecifics).
(TIF)

S3 Fig Boxplot to Larval development time of *Aedes aegypti* and *Aedes albopictus*. Boxplot to Larval development time (days) of a) *Aedes aegypti* and b) *Aedes albopictus*. A: individual alone; B: Larva + 1 conspecific; C: Larva + 2 conspecific; D: Larva + 1 heterospecific; E: Larva + 1 conspecific +1 heterospecific; F: Larva + 2 heterospecifics).
(TIF)

S4 Fig Boxplot to Adult survival at starvation of *Aedes aegypti* and *Aedes albopictus*. Boxplot to Adult survival at starvation (days) of a) *Aedes aegypti* and b) *Aedes albopictus*. A: individual alone; B: Larva + 1 conspecific; C: Larva + 2 conspecifics; D: Larva + 1 heterospecific; E: Larva + 1 conspecific +1 heterospecific; F: Larva + 2 heterospecifics.
(TIF)

S5 Fig Boxplot to Wing length (mm) of *Aedes aegypti* and *Aedes albopictus*. Boxplot to Wing length (mm) of a) *Aedes aegypti* and b) *Aedes albopictus*. A: individual alone; B: Larva + 1 conspecific; C: Larva + 2 conspecifics; D: Larva + 1 heterospecific; E: Larva + 1 conspecific +1 heterospecific; F: Larva + 2 heterospecifics.
(TIF)
