# Supplementary figures and images for: Better to Be in Bad Company than to Be Alone? Aedes Vectors Respond Differently to Breeding Site Quality in the Presence of Others
Source: PLoS One. 2015 Aug 5;10(8):e0134450. doi: 10.1371/journal.pone.0134450 (PMC4526638; doi:10.1371/journal.pone.0134450)

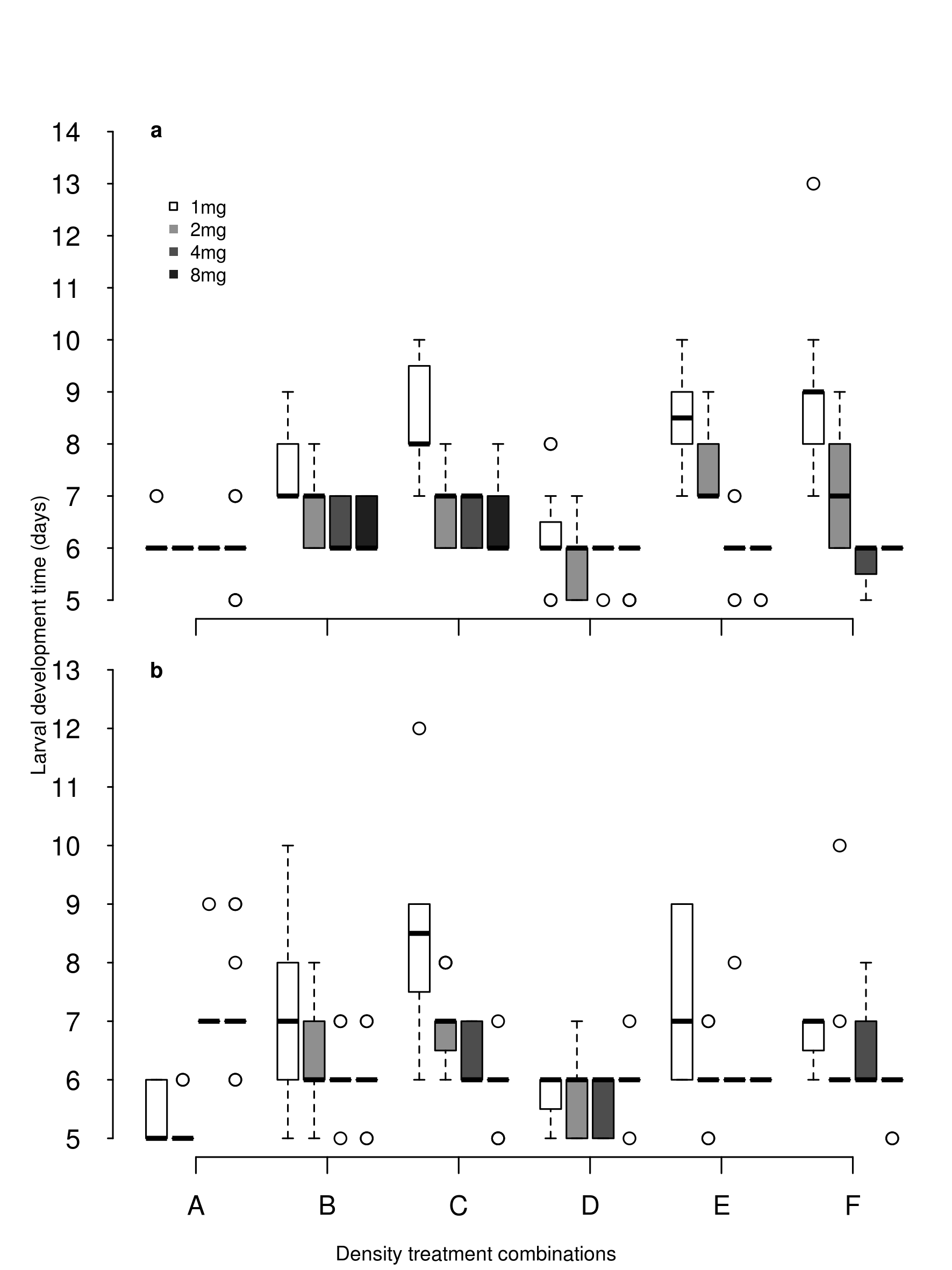

Supplement: S1 Fig — Aedes aegypti survival analysis—Line 1: comparison between individuals kept alone (A: individual alone) with those kept at the presence of one competitor (B: Larva + 1 conspecific; D: Larva + 1 heterospecific). Line 2: comparison between individuals kept alone (A: individual alone) with those kept at the presence of two competitors (C: Larva + 2 conspecifics; E: Larva + 1 conspecific +1 heterospecific; F: Larva + 2 heterospecifics). (TIF) [file pone.0134450.s002.tif]

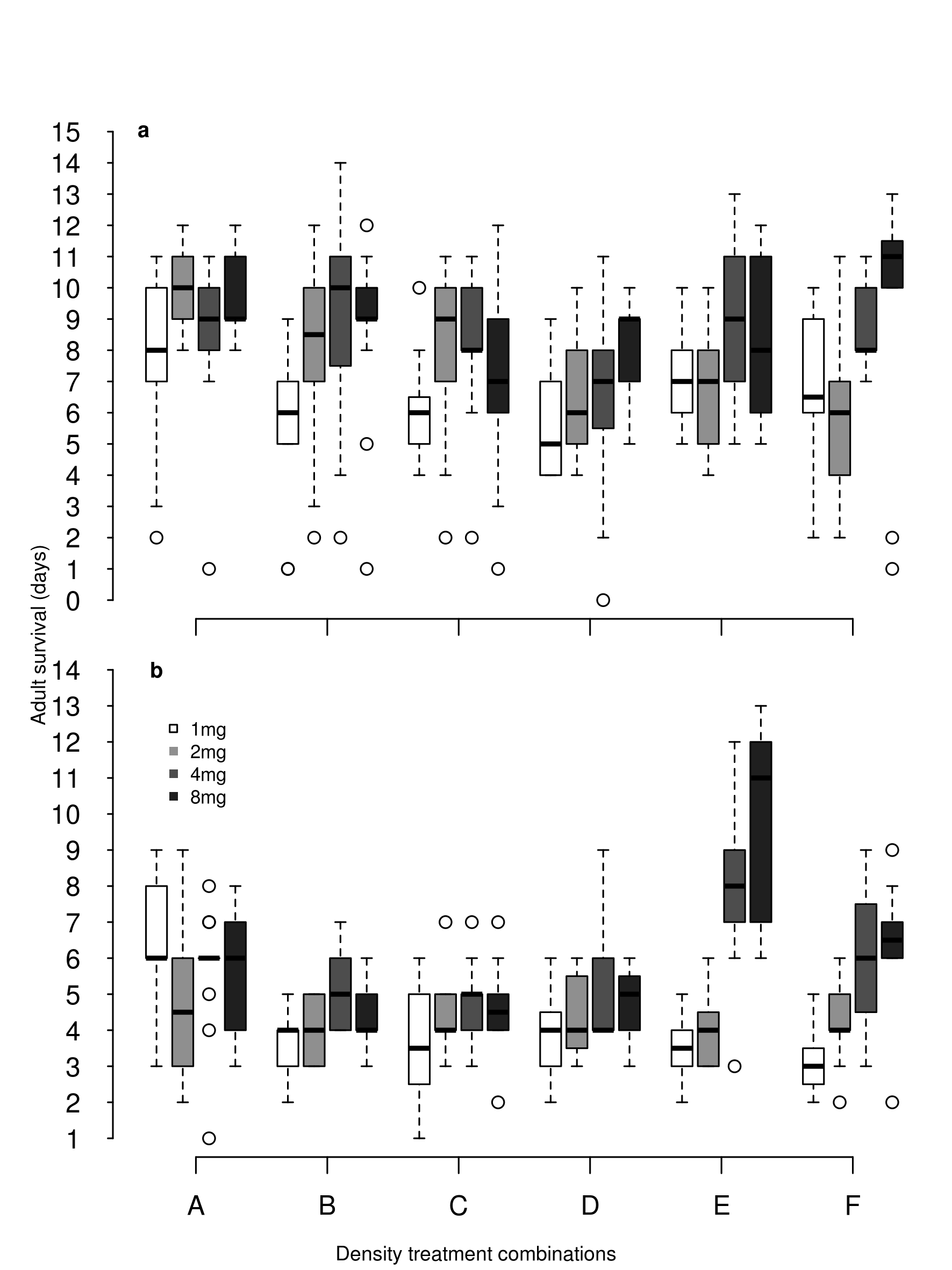

Supplement: S2 Fig — Aedes albopictus survival analysis—Line 1: comparison between individuals kept alone (A: individual alone) with those kept at the presence of one competitor (B: Larva + 1 conspecific; D: Larva + 1 heterospecific). Line 2: comparison between individuals kept alone (A: individual alone) with those kept at the presence of two competitors (C: Larva + 2 conspecifics; E: Larva + 1 conspecific +1 heterospecific; F: Larva + 2 heterospecifics). (TIF) [file pone.0134450.s003.tif]

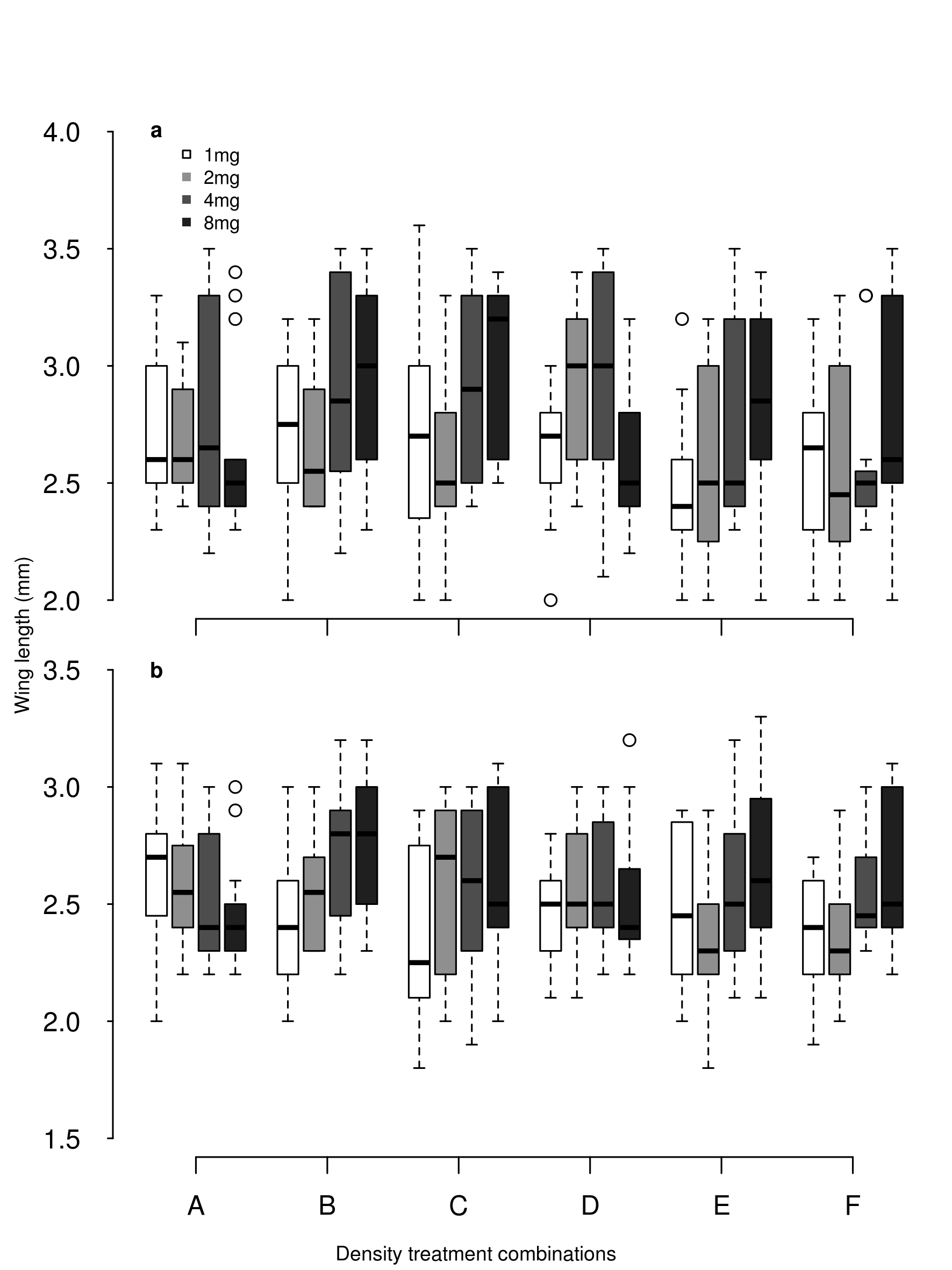

Supplement: S3 Fig — Boxplot to Larval development time (days) of a) Aedes aegypti and b) Aedes albopictus. A: individual alone; B: Larva + 1 conspecific; C: Larva + 2 conspecific; D: Larva + 1 heterospecific; E: Larva + 1 conspecific +1 heterospecific; F: Larva + 2 heterospecifics). (TIF) [file pone.0134450.s004.tif]

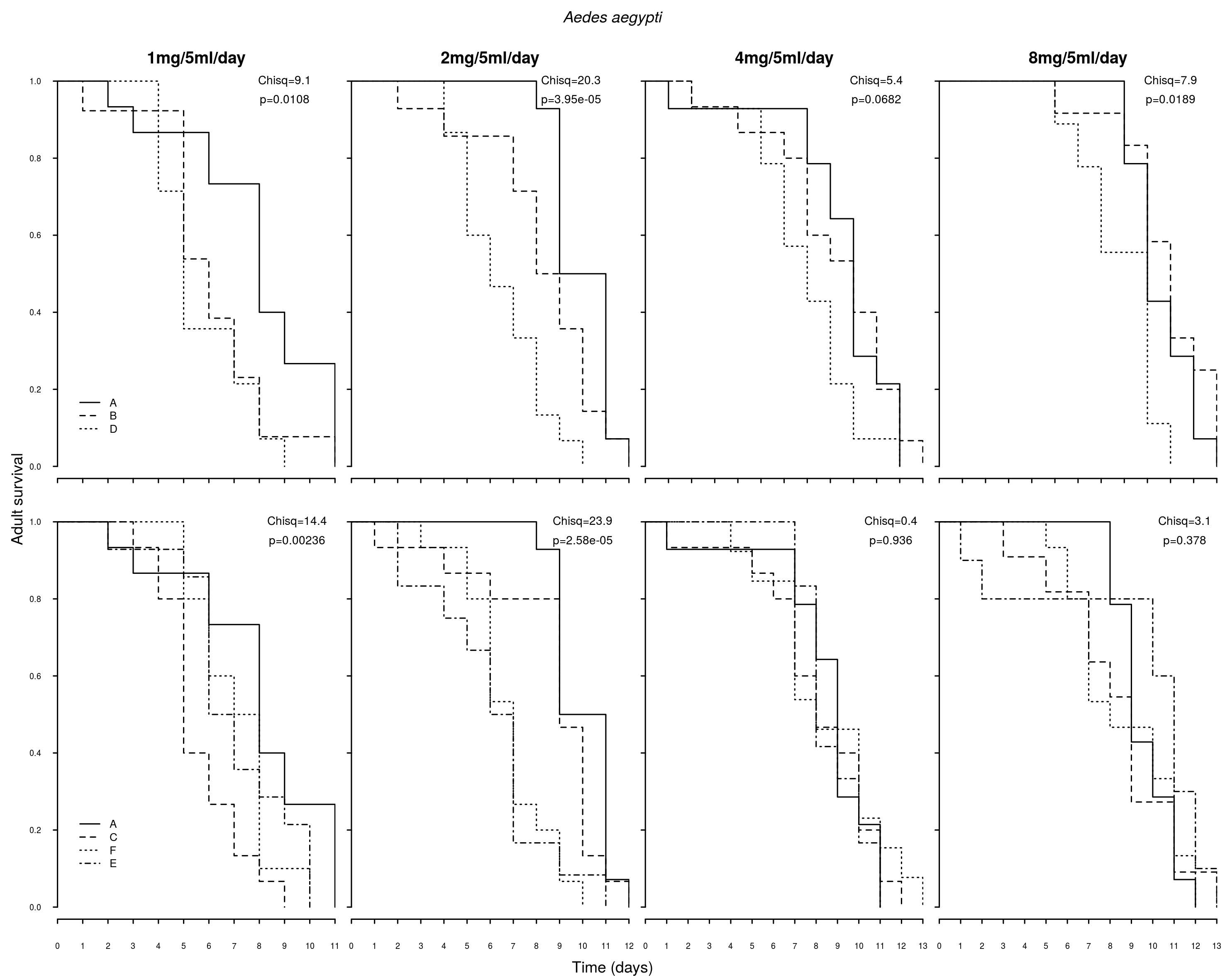

Supplement: S4 Fig — Boxplot to Adult survival at starvation (days) of a) Aedes aegypti and b) Aedes albopictus. A: individual alone; B: Larva + 1 conspecific; C: Larva + 2 conspecifics; D: Larva + 1 heterospecific; E: Larva + 1 conspecific +1 heterospecific; F: Larva + 2 heterospecifics. (TIF) [file pone.0134450.s005.tif]

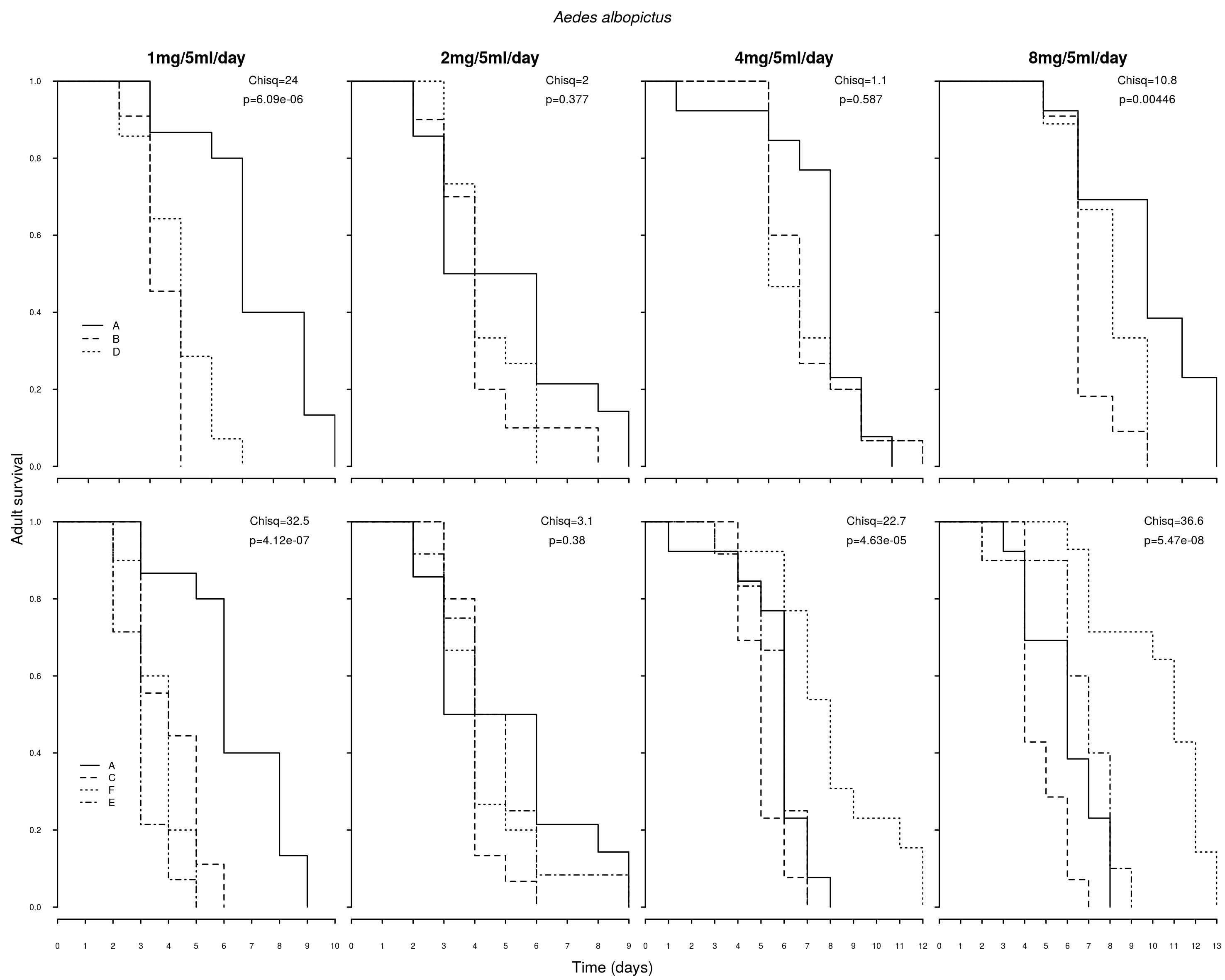

Supplement: S5 Fig — Boxplot to Wing length (mm) of a) Aedes aegypti and b) Aedes albopictus. A: individual alone; B: Larva + 1 conspecific; C: Larva + 2 conspecifics; D: Larva + 1 heterospecific; E: Larva + 1 conspecific +1 heterospecific; F: Larva + 2 heterospecifics. (TIF) [file pone.0134450.s006.tif]
